# Autograft versus allograft in anterior cruciate ligament reconstruction: A meta-analysis with trial sequential analysis

**DOI:** 10.1097/MD.0000000000004936

**Published:** 2016-09-23

**Authors:** Shun-Li Kan, Zhi-Fang Yuan, Guang-Zhi Ning, Bo Yang, Hai-Liang Li, Jing-Cheng Sun, Shi-Qing Feng

**Affiliations:** a Department of Orthopaedics, Tianjin Medical University General Hospital; b School of Nursing, Tianjin Medical University, Tianjin, China.

**Keywords:** allograft, anterior cruciate ligament reconstruction, autograft, meta-analysis, trial sequential analysis

## Abstract

**Background:**

Anterior cruciate ligament (ACL) reconstruction is considered as the standard surgical procedure for the treatment of ACL tear. However, there is a crucial controversy in terms of whether to use autograft or allograft in ACL reconstruction. The purpose of this meta-analysis is to compare autograft with allograft for patients undergoing ACL reconstruction.

**Methods:**

PubMed, EMBASE, and the Cochrane Library were searched for randomized controlled trials that compared autograft with allograft in ACL reconstruction up to January 31, 2016. The relative risk or mean difference with 95% confidence interval was calculated using either a fixed- or random-effects model. The risk of bias for individual studies according to the Cochrane Handbook. The trial sequential analysis was used to test the robustness of our findings and get more conservative estimates.

**Results:**

Thirteen trials were included, involving 1636 participants. The results of this meta-analysis indicated that autograft brought about lower clinical failure, better overall International Knee Documentation Committee (IKDC) level, better pivot-shift test, better Lachman test, greater Tegner score, and better instrumented laxity test (*P* < 0.05) than allograft. Autograft was not statistically different from allograft in Lysholm score, subjective IKDC score, and Daniel 1-leg hop test (*P* > 0.05). Subgroup analyses demonstrated that autograft was superior to irradiated allograft for patients undergoing ACL reconstruction in clinical failure, Lysholm score, pivot-shift test, Lachman test, Tegner score, instrumented laxity test, and subjective IKDC score (*P* < 0.05). Moreover, there were no significant differences between autograft and nonirradiated allograft.

**Conclusions:**

Autograft is superior to irradiated allograft for patients undergoing ACL reconstruction concerning knee function and laxity, but there are no significant differences between autograft and nonirradiated allograft. However, our results should be interpreted with caution, because the blinding methods were not well used.

## Abstract 2

Supplemental Digital Content is available in the text

## 1 Introduction

Anterior cruciate ligament (ACL) tear is a common injury, which occurs in about 250,000 people in the United States each year.^[1,2]^ One large New Zealand study found an incidence of 36.9 injuries per 100,000 person-years.^[3]^ It has been proved that a torn ACL cannot heal with conservative management and repair alone.^[4,5]^ Accordingly, ACL reconstruction is considered as the standard surgical procedure for the treatment of ACL tear. The graft used for ACL reconstruction includes autograft and allograft (irradiated and nonirradiated). However, there is a crucial controversy in terms of whether to use autograft or allograft in ACL reconstruction.^[6,7]^ Although autograft has the advantages of earlier incorporation and no rejection or disease transmission, it may result in donor-site morbidity. The advantages of allograft include the availability of numerous grafts, avoidance of donor-site morbidity, shorter operation time, and shorter rehabilitation time.^[8–10]^ However, its major disadvantages are higher graft cost, disease transmission, delayed graft incorporation, and worse functional outcome.^[11]^ Gamma irradiation has been used to prevent infection caused by allograft. However, several studies have indicated that this sterilization method considerably change the biomechanical and biochemical properties of allograft.^[12,13]^

Previous systematic review and meta-analyses^[14–17]^ comparing autograft with allograft in ACL reconstruction had controversial results. Because almost all of the studies pooled the data from randomized controlled trials and observational studies and the data coming from observational studies are subject to bias, the reliability of the results was compromised. Recently, randomized controlled trials in terms of this issue have reported conflicting results. We performed this meta-analysis of randomized controlled trials to compare autograft with allograft in ACL reconstruction. Furthermore, we also used trial sequential analysis (TSA) to test the robustness of our findings and get more conservative estimates.

## 2 Materials and methods

### 2.1 Search strategy and study selection

PubMed, EMBASE, and the Cochrane Library were searched for randomized controlled trials that compared autograft with allograft in ACL reconstruction up to January 31, 2016. We used the combination of MeSH terms and text words in the electronic search. The search terms regarding to ACL reconstruction were combined with terms related to both autograft and allograft. The details of the search strategies are shown in Supplementary Table S1. There were no language and publication status restrictions. We also manually examined the systematic reviews, meta-analyses, and any other articles included in our meta-analysis for additional relevant articles. Titles and abstracts were screened, and the full text of potentially eligible studies was screened independently by 2 reviewers.

### 2.2 Eligibility criteria

#### 2.2.1 Participants

Trials with adult patients undergoing primary ACL reconstruction were included. Patients with a revision ACL reconstruction were excluded.

#### 2.2.2 Interventions and comparisons

Trials with ACL reconstruction comparing autograft with allograft were included. The type of allograft or autograft was not restricted.

#### 2.2.3 Outcomes

Studies were qualified when at least one of the following outcomes were described: clinical failure, overall International Knee Documentation Committee (IKDC) level, Lysholm score, pivot-shift test, Lachman test, Tegner score, instrumented laxity test, subjective IKDC score, and Daniel 1-leg hop test.

#### 2.2.4 Study design

Only randomized controlled trials were included in our study.

### 2.3 Data extraction and outcome measures

Two independent investigators performed data extraction. For each included article, the following information was extracted: details of methodology, participants, intervention characteristic, follow-up interval, and outcomes. If the means, standard deviations or standard error of the means were not available in the text of articles, we extracted data from the diagrams and tables, if available.^[18]^ Disagreements were resolved by discussion.

The primary outcome measure of interest was clinical failure (including revision surgery, graft rupture, +2 pivot shift or higher, and side-to-side arthrometer difference >5 mm^17^). The secondary outcome measures included overall IKDC level, Lysholm score, pivot-shift test, Lachman test, Tegner score, instrumented laxity test, subjective IKDC score, and Daniel 1-leg hop test. Examination of knee laxity included the Lachman test, the pivot shift test, and the instrumented laxity test. Functional tests included overall IKDC level and the Daniel one-leg hop tests. The Tegner score and the Lysholm score were also used to assess patient's activity level and knee function.

### 2.4 Risk of bias assessment

Two reviewers independently evaluated the risk of bias for individual studies according to the Cochrane Handbook.^[18]^ The parts of assessment consisted of random sequence generation, allocation concealment, blinding of participants and personnel, blinding of outcome assessors, incomplete outcome data, selective outcome reporting, and other bias (baseline balance and fund). All of the fields were determined as low risk of bias, high risk of bias, or unclear risk of bias.

### 2.5 Quality of evidence assessment

The quality of evidence for all the outcomes was rated according to the Grading of Recommendations Assessment, Development and Evaluation (GRADE) methodology.^[19]^ The assessment was based on risk of bias, inconsistency, indirectness, imprecision, and publication bias.^[19,20]^ Each outcome was rated as high, moderate, low, or very low. Summary tables were constructed using GRADE Pro version 3.6 (GRADE Working Group).

### 2.6 Statistical analysis

We calculated relative risk (RR) with 95% confidence interval (CI) for dichotomous outcomes and mean difference (MD) with corresponding 95% CI for continuous outcomes. The I^2^ statistic was used to quantify heterogeneity, with I^2^ greater than 50% suggesting significant heterogeneity.^[21]^ The random-effects model was used if there was significant heterogeneity. Otherwise, the fix-effects model was used. Based on whether the graft was irradiated in the allograft group (irradiated vs nonirradiated) and the type of graft in the allograft group (soft tissue vs bone-patellar tendon-bone [BPTB]), we conducted subgroup analyses. We performed sensitivity analyses using odds ratio or standardized mean difference, and excluding the largest trial, the most weighted trial, and the trial with high risk of bias. Furthermore, we conducted meta-regression analyses to evaluate the potential influence of mean age and male ratio on the primary outcome. Egger linear regression test and funnel plots were used to test the publication bias when more than ten publications were included. *P* values less than 0.05 denoted significant differences. Review Manager version 5.3 (The Nordic Cochrane Centre, The Cochrane Collaboration, Copenhagen, 2014) and Stata version 12.0 (Stata Corp, College Station, TX) were used for the statistical analyses.

### 2.7 Trial sequential analysis

In a meta-analysis, the risk of false positive errors (type I error) may arise. This phenomenon may result from random errors when a small number of studies and participants is analyzed^[22–24]^ and repetitive statistical testing of the accumulation of additional data.^[22,25]^ To correct for the incremental risk of type I errors, we used TSA to identify whether the findings of the cumulative meta-analysis were dependable and conclusive. TSA combines the required information size with trial sequential monitoring boundaries which adjust the CIs and decrease type I errors.^[25,26]^ When the cumulative z-curve crosses the trial sequential monitoring boundary or enters the futility area, an adequate level of evidence for the anticipated intervention effect may have been reached and no further trials are needed. If the z-curve does not cross any of the boundaries and the required information size has not been reached, the evidence is inadequate to reach a conclusion.

We estimated a diversity-adjusted information size in accordance with the diversity of the intervention effect estimates among the included studies. The TSA was conducted to maintain a type I error of 5% with a power of 80%. In the present meta-analysis, we calculated the required information size using the estimates of the intervention effects of trials with adequate random sequence generation or adequate allocation concealment.^[25,27–29]^ Trial sequential analysis software version 0.9 beta (Copenhagen Trial Unit) (www.ctu.dk/tsa)^[30]^ was used for these analyses.

### 2.8 Ethical statement

As all analyses were grounded on previously published studies, ethical approval was not necessary.

## 3 Results

### 3.1 Study search

**Figure 1:**
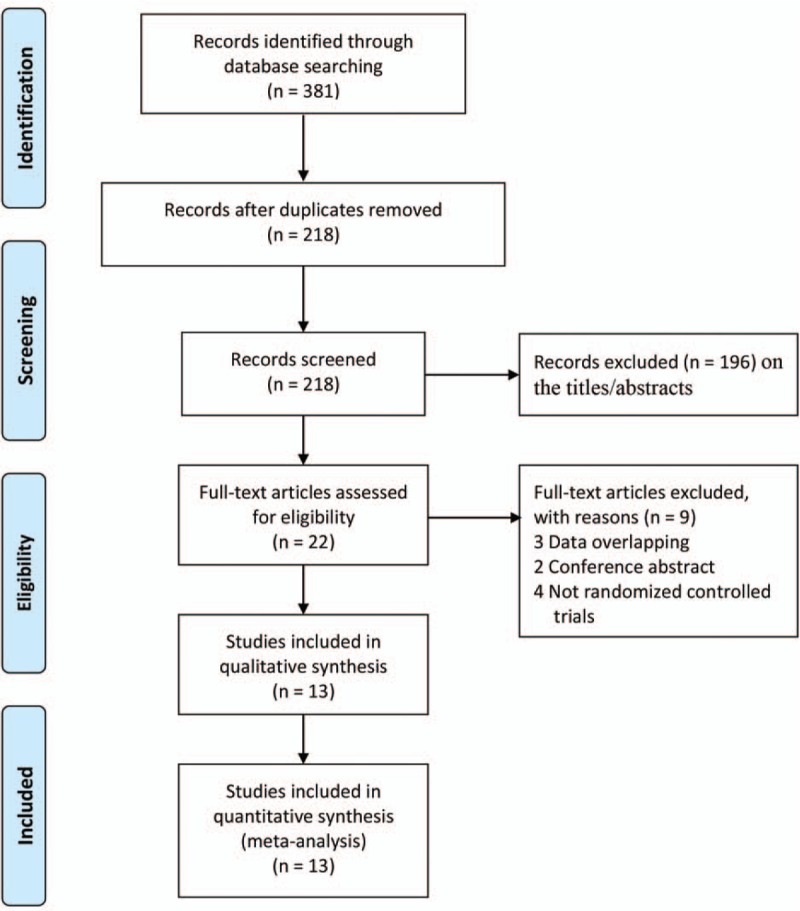
Flow diagram of study selection.

A summary of the study selection process are presented in Fig. 1. Our searches identified 381 records. A total of 359 citations were discarded because they were duplicates or did not fit the eligibility criteria. After the full text of the remaining 22 articles was verified, 13 studies^[31–43]^ were included in the quantitative analysis.

### 3.2 Study characteristics

Table 1 presents the study characteristics. These studies were published between 2002 and 2015. The sample sizes ranged from 31 to 154, with a total of 1636 patients randomly assigned to the autograft group (n = 823) and allograft group (n = 813). Across the trials, the mean age of participants was between 22 and 34.4 years. The participants in most studies were mainly male. Within each study, the autograft group and allograft group used identical surgical approach and fixation method. Likewise, every participant received same postoperative rehabilitation program within each study. The autograft group consisted of hamstring tendon graft^[31,32,34–37,40–43]^ and BPTB graft,^[33,38,39]^ and 7 kinds of grafts, including lower extremity tendon,^[31]^ tibialis posterior tendon,^[32]^ BPTB,^[33,34,38,39]^ tibialis anterior tendon,^[35,36,42]^ free-tendon Achilles,^[37]^ hamstring tendon,^[40,41]^ and tibialis tendon.^[43]^ Five of the 13 included studies reported the use of irradiated allografts.^[31,33,36,38,40]^

**Table 1:**
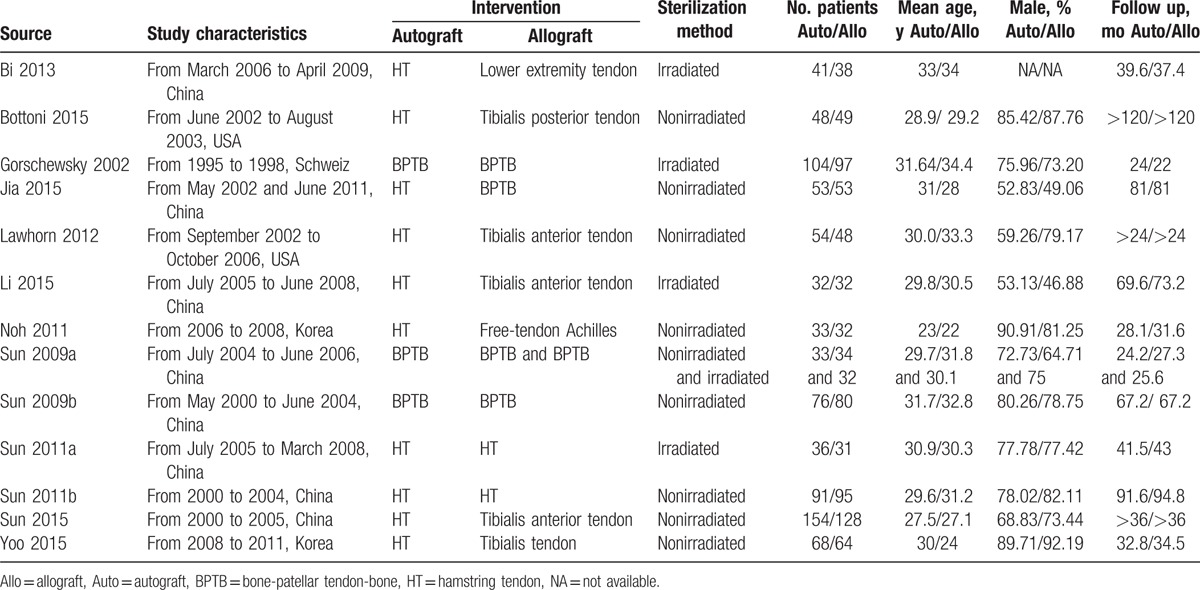
Baseline characteristics of studies included in the meta-analysis.

### 3.3 Risk of bias in the included studies

**Figure 2:**
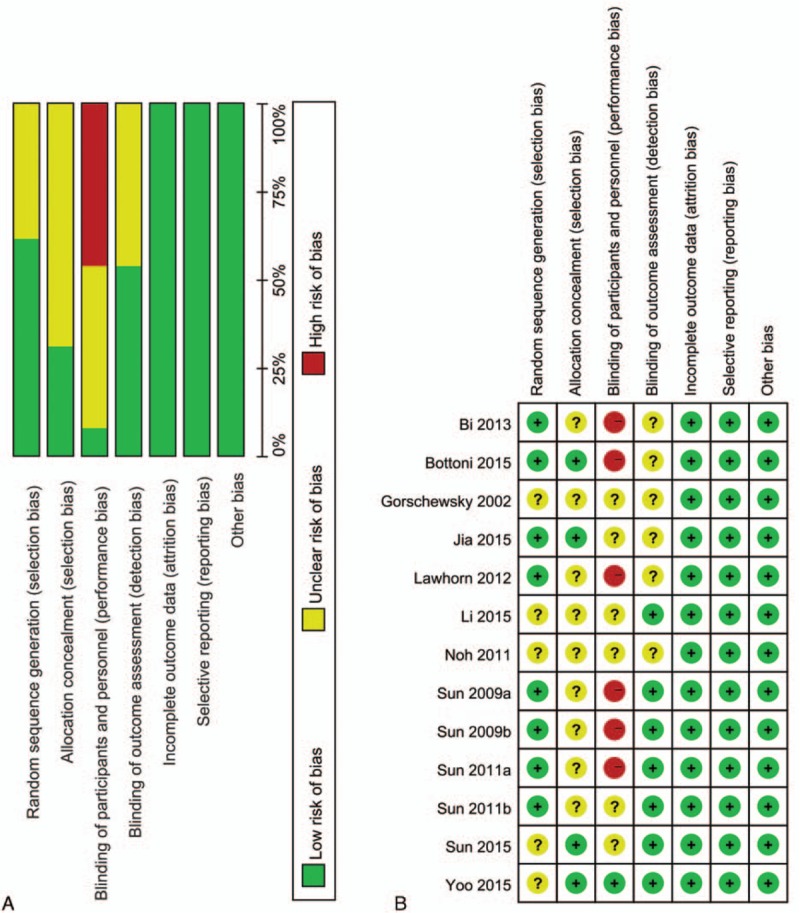
Risk of bias assessment of each included study: (a) Risk of bias graph and (b) risk of bias summary.

The information about the risk of bias for each study is presented in Fig. 2. Six studies^[31,32,35,38–40]^ had a high risk of bias. The other studies were considered to be at unclear risk of bias. Not blinding of the participants resulted in the high risk of bias. Random sequence generation was adequate in eight studies.^[31,32,34,35,38–41]^ Allocation concealment was carried out adequately in 4 studies.^[32,34,42,43]^

### 3.4 Quality of evidence assessment

The GRADE evidence profiles are presented in Supplementary Table S2. The GRADE level of evidence was moderate for clinical failure, overall IKDC level, Tegner score; low for Lysholm score, pivot-shift test, instrumented laxity test, subjective IKDC score, Daniel 1-leg hop test; and very low for Lachman test.

### 3.5 Primary outcome

**Figure 3:**
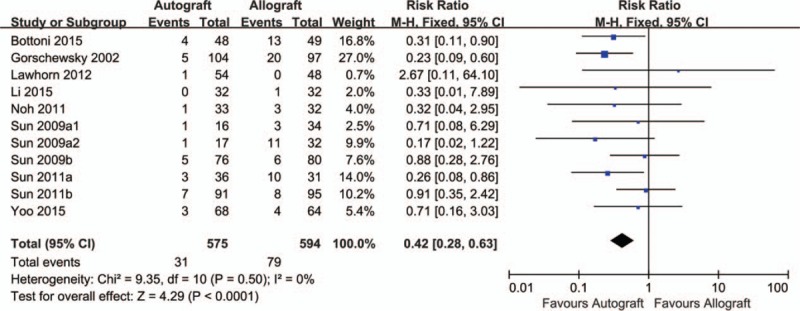
Results of meta-analysis of outcomes between autograft and allograft for clinical failure.

**Figure 4:**
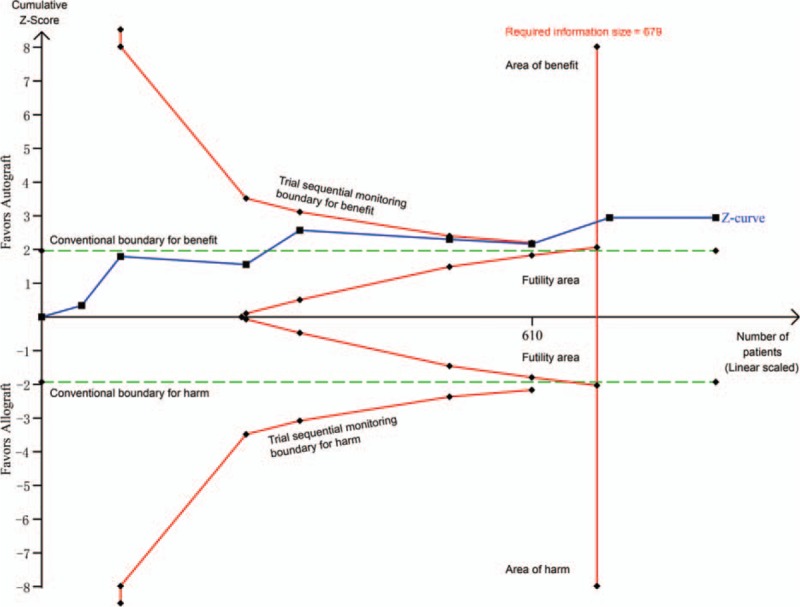
Trial sequential analysis of 8 trials comparing autograft with allograft for clinical failure. Trial sequential analysis of 8 trials (black square fill icons) illustrating that the cumulative z-curve crossed both the traditional boundary and the trial sequential monitoring boundary and the required information size had been reached, suggesting further trials were not necessary and the inferences would not be changed. A diversity adjusted required information size of 679 patients was calculated using α = 0.05 (2 sided), β = 0.20 (power 80%), a RR reduction of 49.76% based on trials with adequate allocation concealment, and an event proportion of 12.70% in the control arm. *X*-axis: the number of patients randomized; *Y*-axis: the cumulative Z-score; Horizontal green dotted lines: conventional boundaries (upper for benefit, Z-score = 1.96, lower for harm, Z-score = −1.96, 2-sided *P* = 0.05); Sloping red full lines with black square fill icons: trial sequential monitoring boundaries calculated accordingly; Blue full line with black square fill icons: Z-curve; Vertical red full line: required information size calculated accordingly.

Ten trials (11 comparisons) including 1169 patients reported data on clinical failure.^[32,33,35–41,43]^ Compared with allograft, autograft significantly reduced clinical failure (RR = 0.42, 95% CI 0.28–0.63, *P* < 0.0001; I^2^ = 0%; Fig. 3). The cumulative z-curve crossed both the traditional boundary and the trial sequential monitoring boundary and the required information size had been reached, suggesting further trials were not necessary and the inferences would not be changed (Fig. 4). Meta-regression analyses indicated no effect of mean age and male ratio in decreasing clinical failure (Supplementary Figures S1 and S2).

### 3.6 Secondary outcomes

Compared with allograft, autograft for ACL reconstruction increased overall IKDC level (RR = 1.03, 95% CI 1.00–1.06, *P* = 0.03; Table 2), pivot-shift test (RR = 1.06, 95% CI 1.02–1.11, P = 0.008; Table 2), Lachman test (RR = 1.14, 95% CI 1.02–1.28, *P* = 0.02; Table 2), and Tegner score (MD = 0.26, 95% CI 0.06–0.45, *P* = 0.01; Table 2). Autograft significantly decreased instrumented laxity test (MD = −0.74, 95% CI −1.15 to −0.32, *P* = 0.0005; Table 2) compared with allograft. Autograft was not significantly different from allograft in terms of the Lysholm score (MD = 0.27, 95% CI −0.79 to 1.32, *P* = 0.62; Table 2), subjective IKDC score (MD = 1.51, 95% CI −0.13 to 3.14, *P* = 0.07; Table 2), and Daniel 1-leg hop test (RR = 0.99, 95% CI 0.91–1.07, *P* = 0.81; Table 2).

**Table 2:**
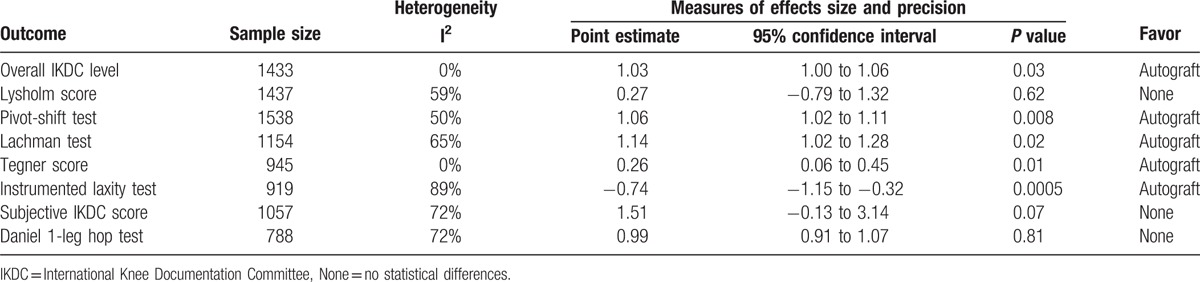
The pooled results of meta-analysis.

### 3.7 Subgroup analyses, sensitivity analyses, and publication bias

The findings of subgroup analyses are presented in Supplementary Table S3. Autograft performed better pertaining to clinical failure, Lysholm score, pivot-shift test, Lachman test, Tegner score, instrumented laxity test, and subjective IKDC score than irradiated allograft and no significant differences were found between autograft and nonirradiated allograft. On the other hand, autograft gained better outcomes than the soft tissue allograft in terms of clinical failure, Tegner score, and instrumented laxity test and achieved lower subjective IKDC score than the BPTB allograft.

There were statistically significant differences between autograft and allograft in pivot-shift test except excluding the high risk of bias trials by Bi et al^[31]^ or Sun et al^[38,39]^ and in Tegner score except that the trial with a high risk of bias by Bi et al^[31]^ was excluded. Subjective IKDC score in the autograft group was significantly different from that in the allograft group when excluding the largest trial by Sun et al^[42]^ (Supplementary Table S4).

The Egger linear regression test and funnel plots were used for 6 results. The funnel plots were visually assessed and did reveal some asymmetry; however, no evidence of publication bias was achieved by the Egger linear regression test for clinical failure (*P* = 0.72, Supplementary Figure S3), overall IKDC level (*P* = 0.14, Supplementary Figure S4), Lysholm score (*P* = 0.06, Supplementary Figure S5), and Tegner score (*P* = 0.27, Supplementary Figure S6). The Egger linear regression test revealed significant publication bias for pivot-shift test (*P* = 0.003, Supplementary Figure S7) and Lachman test (*P* = 0.004, Supplementary Figure S8).

## 4 Discussion

The present meta-analysis systematically reviewed all the evidence and found that autograft significantly decreased clinical failure for patients undergoing ACL reconstruction. This finding was consistent in most subgroup analyses and was verified by sensitivity analyses, meta-regression analyses, and TSA; autograft further reduced instrumented laxity test. In addition, autograft significantly increased overall IKDC level, pivot-shift test, Lachman test, and Tegner score; there were no significant differences between autograft and allograft for Lysholm score, subjective IKDC score and Daniel 1-leg hop test; and subgroup analyses demonstrated that autograft is superior to irradiated allograft. The GRADE level of evidence was moderate for clinical failure, overall IKDC level, and Tegner score; low for Lysholm score, pivot-shift test, instrumented laxity test, subjective IKDC score, and Daniel 1-leg hop test; and very low for Lachman test.

Clinical failure rate after ACL reconstruction varied in the literatures, with higher rate of clinical failure for allograft than autograft. For example, Prodromos et al^[15]^ reported a 5% failure rate for autograft compared with 14% for allograft in their study. Kaeding et al^[44]^ reported a 3.5% failure rate for autograft versus 8.9% for allograft in their cohort. There was significantly less clinical failure in the autograft group in our meta-analysis. In a recent meta-analysis, Prodromos et al,^[15]^ Yao et al,^[45]^ and Zeng et al^[17]^ found that autograft gained significantly less clinical failure compared with allograft. Although the finding was consistent with ours, our study included all the available evidence, which generally coincided and further strengthened earlier findings of previous meta-analyses. Additionally, the TSA was used in this meta-analysis to generate more conservative estimates. The finding of TSA indicated that the present research established ample and convincing evidence. A previous study by Hu et al^[14]^ revealed that no significant difference existed between autograft and allograft in terms of clinical failure. The reason for the different finding between Hu's meta-analysis and ours may be that Hu and colleagues included several nonrandomized controlled trials. Recent studies have demonstrated that younger patients undergoing ACL allograft reconstruction have increased rates of graft failure.^[44,46]^ But our meta-regression analyses shown that no effect of mean age on clinical failure. The different finding may result from the fact that randomized controlled trials enrolled an older and less active patient population than the nonrandomized controlled trials. Further trials with patients in their late teens and early 20s would be required to verify the results of ACL reconstruction with autograft compared with allograft.

The advantage of irradiated allograft was a decreased risk of disease transmission, but researches have shown that irradiation decreased the biomechanical properties of the allograft.^[47,48]^ However, a recent trial with low-dose (1.0- to 1.2-Mrad) gamma irradiation of BPTB allografts showed reduced graft stiffness by 20% without any change in biomechanical properties.^[49]^ Clinical trials have yielded diverse findings in terms of whether irradiated allograft led to higher rates of clinical failure. Rappe et al^[50]^ observed a 33% failure rate for irradiated allograft versus 2.4% for nonirradiated allograft. And our study also demonstrated that irradiated allograft rather than nonirradiated allograft significantly increased clinical failure compared with autograft. Such findings suggested that nonirradiated allograft can be regarded as a substitute to autograft for ACL reconstruction. McGuire and Hendricks,^[51]^ likewise, suggested no significant difference was found in local and systemic immune responses with respect to unfavorably influencing graft healing and clinical outcomes between autograft and nonirradiated allograft. Contrariwise, Rihn et al^[52]^ found irradiation had no unfavorable effect on clinical outcome in ACL reconstruction with allograft. Whether irradiation was used or not was a major factor that influenced the results after ACL reconstruction with allograft.

Autograft achieved better outcomes pertaining to clinical failure, Lysholm score, pivot-shift test, Lachman test, Tegner score, instrumented laxity test, and subjective IKDC score than irradiated allograft. The change of biomechanical properties by irradiation may be harmful to graft function and influence the clinical results when such graft is used for the ACL reconstruction, which may be a possible reason for the findings.

In clinical decision making, healthcare providers should consider not only the efficacy of autograft and allograft but also patients’ age, the costs, and whether irradiation should be used for allograft. Policy makers should perform further trials with patients in their late teens and early 20s to verify the results of ACL reconstruction with autograft compared with allograft.

A major advantage of this meta-analysis was that the TSA was used to test the robustness of our findings and get more conservative estimations. In addition, this meta-analysis was conducted based on the best methodology recommended by the Cochrane Collaboration. Furthermore, only randomized controlled trials were included, which could decrease the possibility of inconsistency among different groups and diminish selection bias.

Our analysis also had some limitations. First, all the studies were rated as unclear or high risk of bias, as the method of blinding patients was not known or not used. A blinding method is rarely possible to use on a surgical topic, which is an inherent limitation of conducting randomized trials regarding such topic. Second, differences existed in the inclusion and exclusion criteria among the trials, especially concerning the enrolling patients with concomitant meniscal and/or cartilage injuries at the time of ACL injury. This potentially resulted in different results among studies, although no significant differences were found. Moreover, there were differences in gender ratio of the trials. Most trials were with more male participants, and others had a more even gender ratio. Furthermore, 4 clinical trials conducted by the same authors were included in the present study. It is very possible that these 4 trials were connected, although the included participants did not overlap at all.

## 5 Conclusion

Autograft is superior to irradiated allograft for patients undergoing ACL reconstruction concerning knee function and laxity, but there are no significant differences between autograft and nonirradiated allograft. However, our results should be interpreted with caution, because the blinding methods were not well used.

## Supplementary Material

Supplemental Digital Content
