# Supplementary material for: Autograft versus allograft in anterior cruciate ligament reconstruction: A meta-analysis with trial sequential analysis
Source: Medicine (Baltimore). 2016 Sep 23;95(38):e4936. doi: 10.1097/MD.0000000000004936 (PMC5044918; doi:10.1097/MD.0000000000004936)
Supplement: Supplemental Digital Content [file medi-95-e4936-s001.pdf]

Supplementary Figure S1 Meta-regression analysis of influence of mean age on clinical failure. The circles represent each study. The size of the circle represents the power of the study. The solid line indicates the weighted regression line. RR relative risk

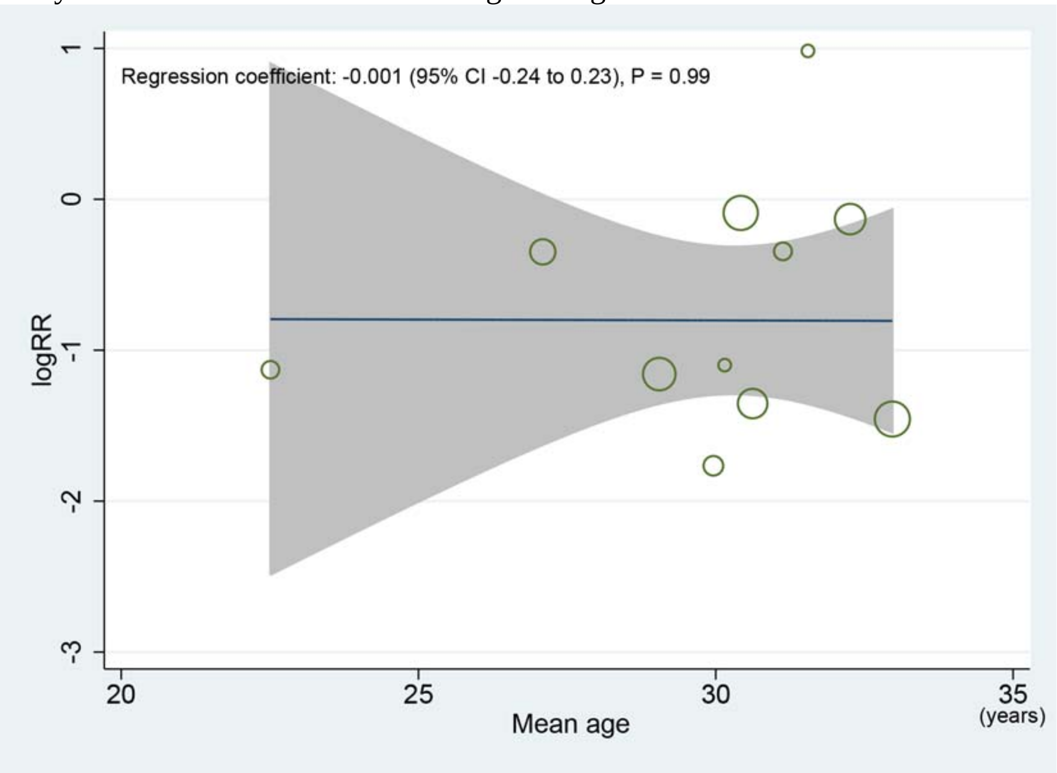

Supplementary Figure S2 Meta-regression analysis of influence of male ratio on clinical failure. The circles represent each study. The size of the circle represents the power of the study. The solid line indicates the weighted regression line. RR relative risk

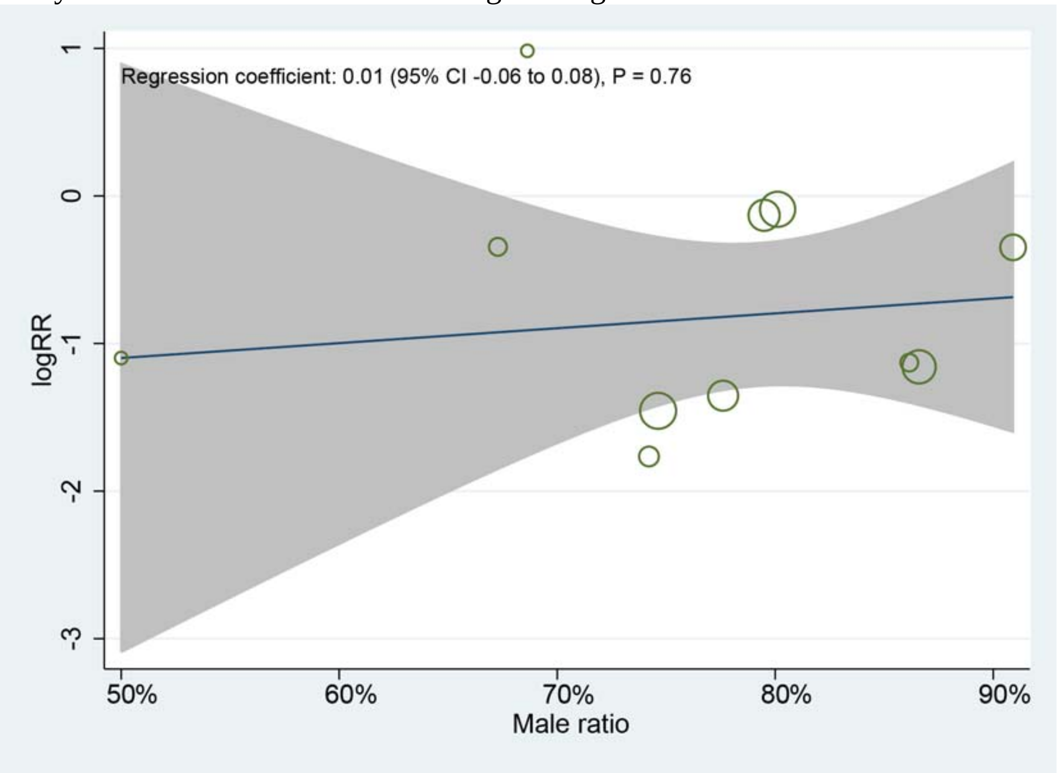

Supplementary Figure S3 Funnel plot of clinical failure of the included studies comparing autograft with allograft. RR relative risk

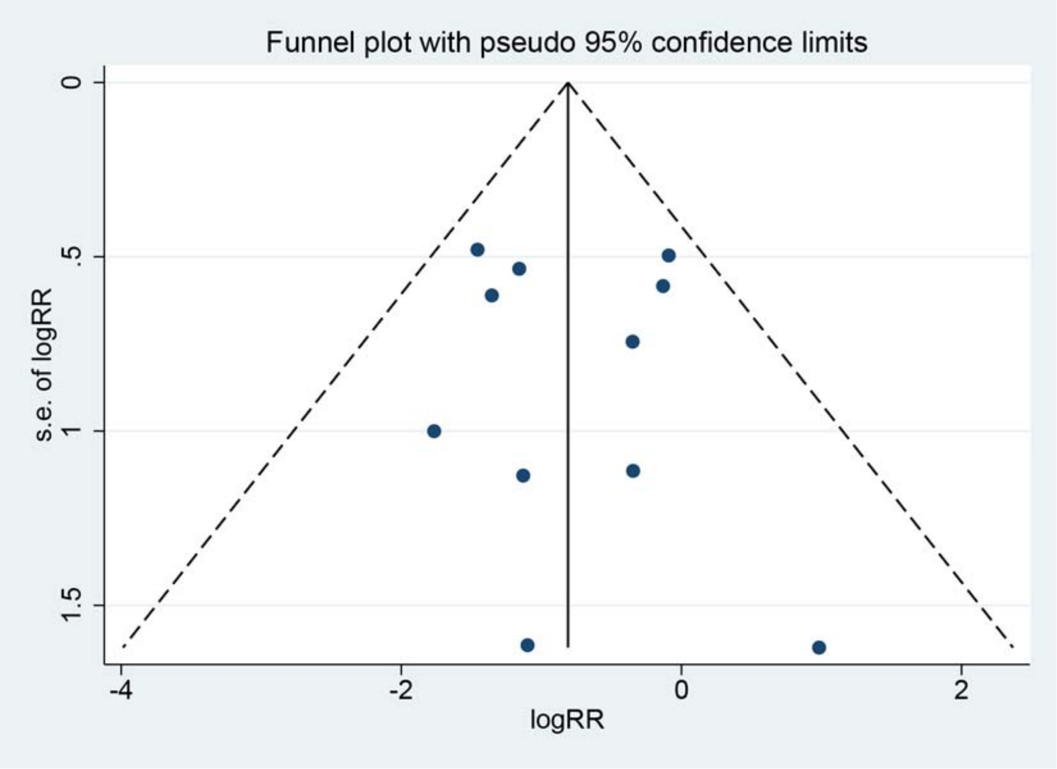

Supplementary Figure S4 Funnel plot of overall IKDC level of the included studies comparing autograft with allograft. RR relative risk, IKDC International Knee Documentation Committee

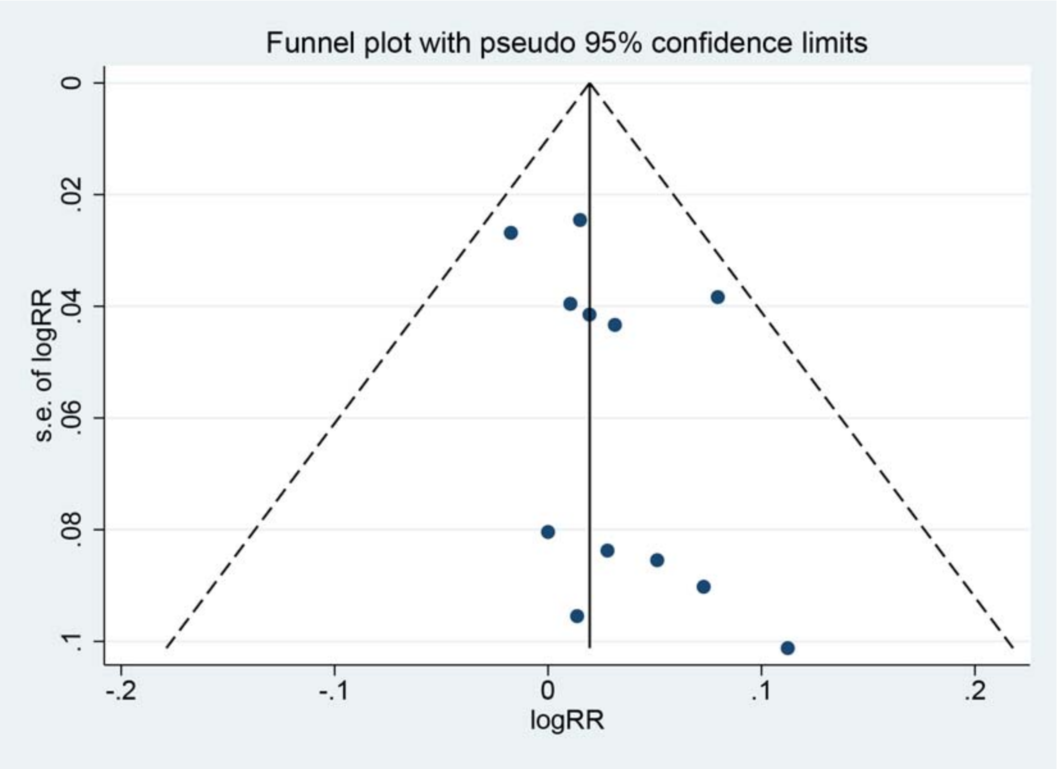

Supplementary Figure S5 Funnel plot of Lysholm score of the included studies comparing autograft with allograft. MD mean difference

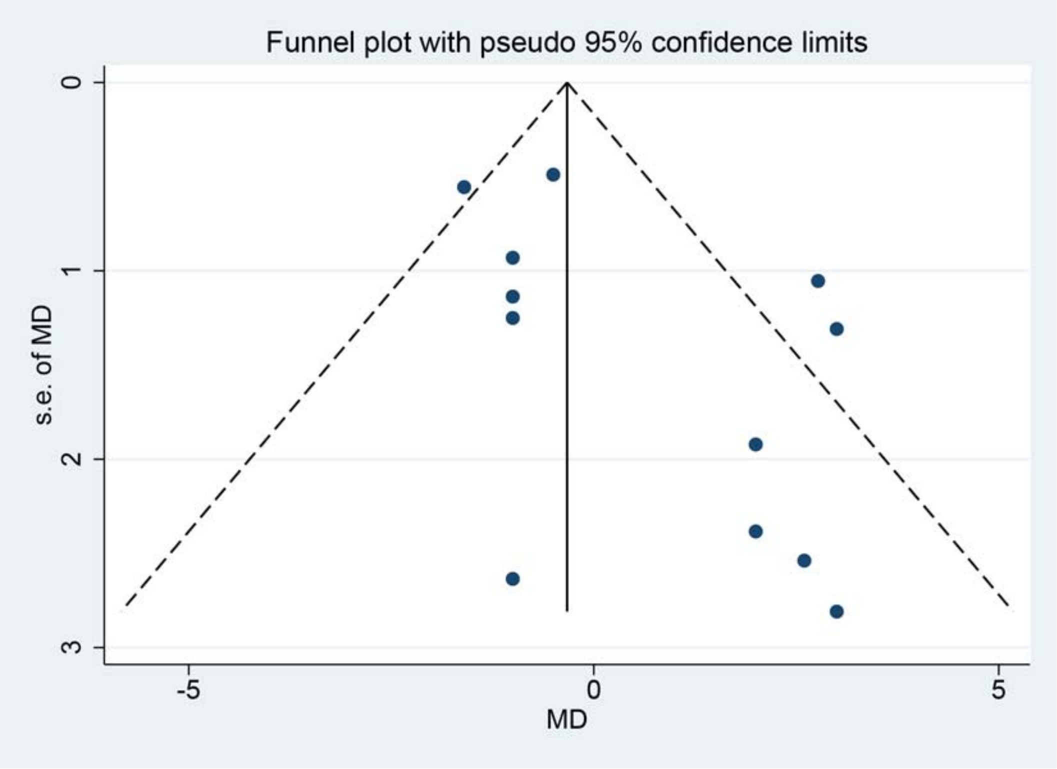

Supplementary Figure S6 Funnel plot of Tegner score of the included studies comparing autograft with allograft. MD mean difference

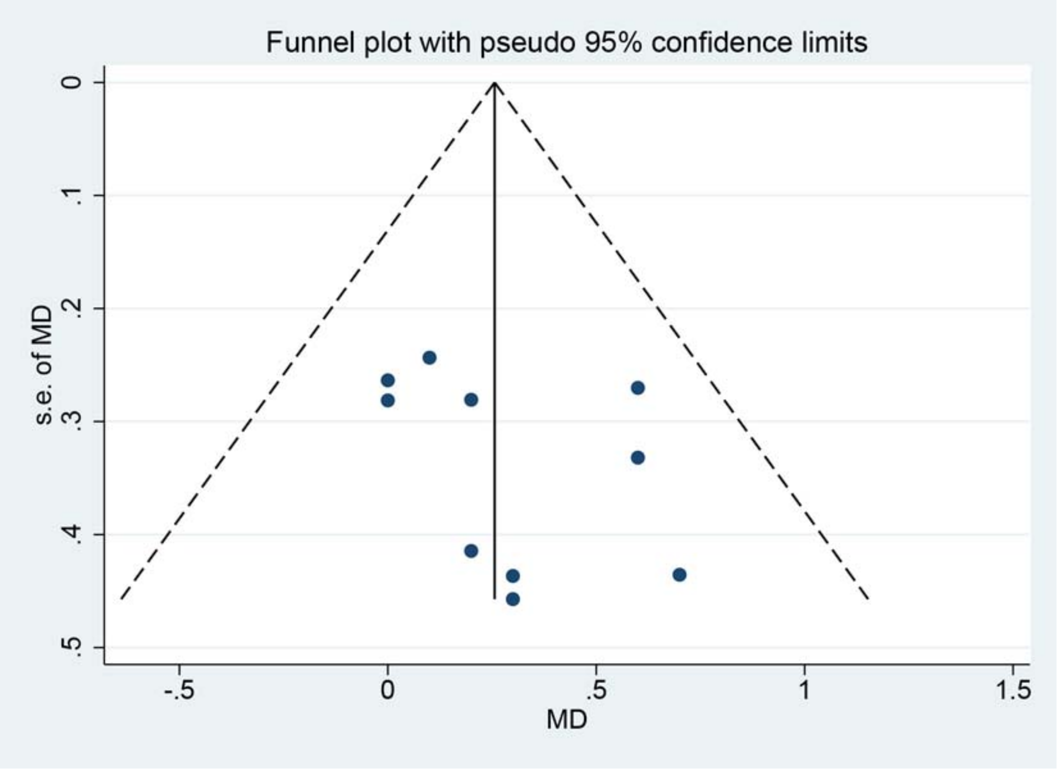

Supplementary Figure S7 Funnel plot of pivot-shift test of the included studies comparing autograft with allograft. RR relative risk

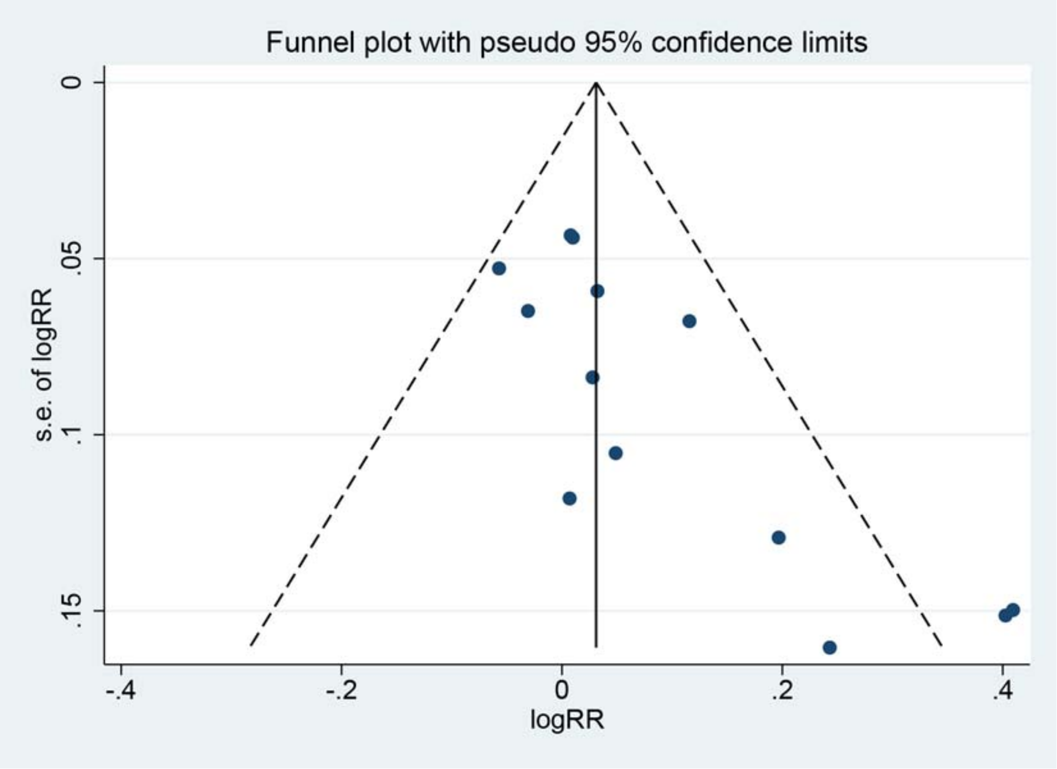

Supplementary Figure S8 Funnel plot of Lachman test of the included studies comparing autograft with allograft. RR relative risk

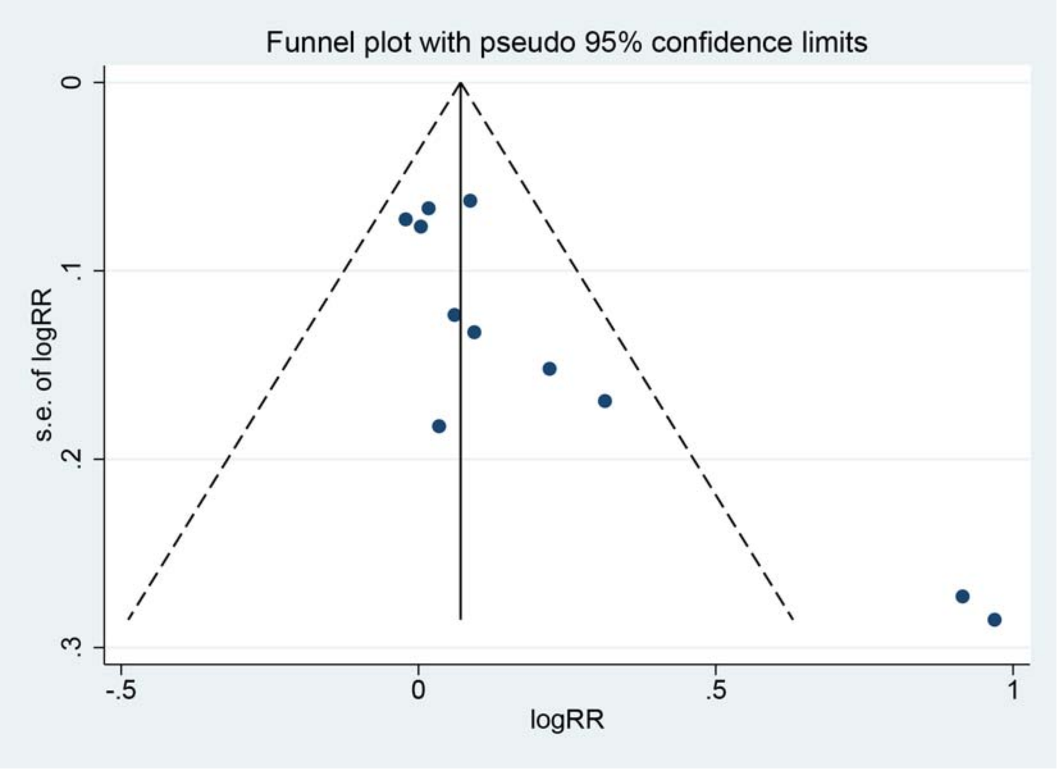

Supplementary Table S1 Search strategies

PubMed
Searched on: January 31, 2016
Results: 159

Search	Query
#1	"Randomized Controlled Trial" [Publication Type] OR "Controlled Clinical Trial" [Publication Type]
#2	(((random*[Title/Abstract]) OR placebo[Title/Abstract]) OR trial*[Title/Abstract]) OR group*[Title/Abstract]
#3	#1 OR #2
#4	"Animals"[Mesh]
#5	"Humans"[Mesh]
#6	#4 NOT #5
#7	#3 NOT #6
#8	"Anterior Cruciate Ligament"[Mesh] OR "Anterior Cruciate Ligament Reconstruction"[Mesh]
#9	((Anterior Cruciate Ligament[Title/Abstract]) OR Anterior Cruciate Ligament Reconstruction[Title/Abstract]) OR ACL[Title/Abstract]
#10	((anterior[Title/Abstract]) AND cruciate*[Title/Abstract]) AND ligament*[Title/Abstract]
#11	#8 OR #9 OR #10
#12	"Transplantation, Autologous"[Mesh]
#13	(((Autologous[Title/Abstract]) OR autogenous[Title/Abstract]) OR autotransplant*[Title/Abstract]) OR autograft*[Title/Abstract]
#14	#12 OR #13
#15	"Transplantation, Homologous"[Mesh]
#16	(((Allograft*[Title/Abstract]) OR homograft*[Title/Abstract]) OR homologous[Title/Abstract]) OR allogeneic*[Title/Abstract]
#17	#15 OR #16
#18	#7 AND #11 AND #14 AND #17

EMBASE
Searched on: January 31, 2016
Results: 187

Search	Query
#1	'randomized controlled trial'/exp
#2	'controlled clinical trial'/exp
#3	random*:ab,ti
#4	placebo:ab,ti
#5	Trial*:ab,ti
#6	Group*:ab,ti
#7	#1 OR #2 OR #3 OR #4 OR #5 OR #6
#8	'animal'/exp
#9	'human'/exp
#10	#8 NOT #9
#11	#7 NOT #10
#12	'anterior cruciate ligament'/exp
#13	'anterior cruciate ligament reconstruction'/exp
#14	'anterior cruciate ligament reconstruction':ab,ti
#15	'anterior cruciate ligament':ab,ti
#16	ACL:ab,ti
#17	anterior:ab,ti
#18	cruciate*:ab,ti
#19	ligament*:ab,ti
#20	#17 AND #18 AND #19
#21	#12 OR #13 OR #14 OR #15 OR #16 OR #20
#22	'autotransplantation'/exp
#23	autologous:ab,ti
#24	autogenous:ab,ti
#25	autotransplant*:ab,ti
#26	autograft*:ab,ti
#27	#22 OR #23 OR #24 OR #25 OR #26
#28	'allotransplantation'/exp

#29	allograft*:ab,ti
#30	homograft*:ab,ti
#31	homologous:ab,ti
#32	allogeneic*:ab,ti
#33	#28 OR #29 OR #30 OR #31 OR #32
#34	#11 AND #21 AND #27 AND #33

CENTRAL
Searched on: January 31, 2016
Results: 35

**Search**	**Query**
#1	MeSH descriptor: [Randomized Controlled Trial] this term only
#2	MeSH descriptor: [Randomized Controlled Trials as Topic] this term only
#3	MeSH descriptor: [Controlled Clinical Trial] this term only
#4	MeSH descriptor: [Controlled Clinical Trials as Topic] this term only
#5	Random*:ti,ab,kw or placebo:ti,ab,kw or trial*:ti,ab,kw or group*:ti,ab,kw (Word variations have been searched)
#6	#1 or #2 or #3 or #4 or #5
#7	MeSH descriptor: [Animals] explode all trees
#8	MeSH descriptor: [Humans] explode all trees
#9	#7 not #8
#10	#6 not #9
#11	MeSH descriptor: [Anterior Cruciate Ligament] explode all trees
#12	MeSH descriptor: [Anterior Cruciate Ligament Reconstruction] explode all trees
#13	Anterior Cruciate Ligament:ti,ab,kw or Anterior Cruciate Ligament Reconstruction:ti,ab,kw or ACL:ti,ab,kw (Word variations have been searched)
#14	anterior:ti,ab,kw and cruciate*:ti,ab,kw and ligament*:ti,ab,kw (Word variations have been searched)
#15	#11 or #12 or #13 or #14
#16	MeSH descriptor: [Transplantation, Autologous] explode all trees
#17	Autologous:ti,ab,kw or autogenous:ti,ab,kw or autotransplant*:ti,ab,kw or

	autograft*:ti,ab,kw (Word variations have been searched)
#18	#16 or #17
#19	MeSH descriptor: [Transplantation, Homologous] explode all trees
#20	Allograft*:ti,ab,kw or homograft*:ti,ab,kw or homologous:ti,ab,kw or allogeneic*:ti,ab,kw (Word variations have been searched)
#21	#19 or #20
#22	#10 and #15 and #18 and #21 in Trials

Supplementary Table S2 GRADE evidence profile

Quality assessment							No of patients		Effect		Quality*	Importance
No of studies	Design	Risk of bias	Inconsistency	Indirectness	Imprecision	Other considerations	Autograft	Allograft	Relative (95% CI)	Absolute		
**Clinical failure**												
11	randomised trials	serious[1]	no serious inconsistency	no serious indirectness	no serious imprecision	none	31/575 (5.4%)	79/594 (13.3%)	RR 0.42 (0.28 to 0.63)	77 fewer per 1000 (from 49 fewer to 96 fewer)	⊕⊕⊕O MODERATE	CRITICAL
								8.8%		51 fewer per 1000 (from 33 fewer to 63 fewer)		
**Overall IKDC level**												
12	randomised trials	serious[1]	no serious inconsistency	no serious indirectness	no serious imprecision	none	683/722 (94.6%)	651/711 (91.6%)	RR 1.03 (1 to 1.06)	27 more per 1000 (from 0 more to 55 more)	⊕⊕⊕O MODERATE	CRITICAL
								90.6%		27 more per 1000 (from 0 more to 54 more)		
**Lysholm score (Better indicated by higher values)**												
12	randomised trials	serious[1]	serious[2]	no serious indirectness	no serious imprecision	none	721	716	-	MD 0.27 higher (0.79 lower to 1.32 higher)	⊕⊕OO LOW	CRITICAL
**Pivot-shift test**												
13	randomised trials	serious[1]	no serious inconsistency	no serious indirectness	no serious imprecision	reporting bias	660/775 (85.2%)	614/763 (80.5%)	RR 1.06 (1.02 to 1.11)	48 more per 1000 (from 16 more to 89 more)	⊕⊕OO LOW	CRITICAL
								81.3%		49 more per 1000 (from 16 more to 89 more)		
**Lachman test**												
11	randomised trials	serious[1]	serious[3]	no serious indirectness	no serious imprecision	reporting bias	450/567 (79.4%)	412/587 (70.2%)	RR 1.14 (1.02 to 1.28)	98 more per 1000 (from 14 more to 197 more)	⊕OOO VERY LOW	CRITICAL
								71.1%		100 more per 1000 (from 14 more to 199 more)		
**Tegner score (Better indicated by higher values)**												
10	randomised trials	serious[1]	no serious inconsistency	no serious indirectness	no serious imprecision	none	458	487	-	MD 0.26 higher (0.06 to 0.45 higher)	⊕⊕⊕O MODERATE	CRITICAL
**Instrumented laxity test (Better indicated by lower values)**												
8	randomised trials	serious[1]	serious[4]	no serious indirectness	no serious imprecision	none	455	464	-	MD 0.74 lower (1.15 to 0.32 lower)	⊕⊕OO LOW	CRITICAL
**Subjective IKDC score (Better indicated by higher values)**												
9	randomised	serious[1]	serious[5]	no serious	no serious	none	523	534	-	MD 1.51 higher (0.13	⊕⊕OO	CRITICAL

	trials			indirectness	imprecision					lower to 3.14 higher)	LOW	
**Daniel 1-leg hop test**												
6	randomised trials	serious[1]	serious[5]	no serious indirectness	no serious imprecision	none	292/345 (84.6%)	342/376 (91%)	RR 0.98 (0.89 to 1.07)	18 fewer per 1000 (from 100 fewer to 64 more)	⊕⊕OO LOW	CRITICAL
								91.6%		18 fewer per 1000 (from 101 fewer to 64 more)		

IKDC = International Knee Documentation Committee.

[1] All the trials were judged to be at high risk of bias or unclear risk of bias.

[2] Significant heterogeneity (I^2 = 59%) was found.

[3] Significant heterogeneity (I^2 = 65%) was found.

[4] Significant heterogeneity (I^2 = 89%) was found.

[5] Significant heterogeneity (I^2 = 72%) was found.

*GRADE Working Group grades of evidence: high quality = further research is very unlikely to change our confidence in the estimate of effect; moderate quality = further research is likely to have an important impact on our confidence in the estimate of effect and may change the estimate; low quality = further research is very likely to have an important impact on our confidence in the estimate of effect and is likely to change the estimate; very low quality = we are very uncertain about the estimate.

Supplementary Table S3 Subgroup analyses of autograft compared with allograft

Subgroup	Sample size	Measures of effects size and precision			Heterogeneity
		Point estimate	95% confidence interval	P value	I^2
**Clinical failure**					
Total	1169	0.42	0.28 to 0.63	< 0.0001	0%
Irradiation used or not					
Irradiated Allograft	381	0.23	0.12 to 0.46	< 0.0001	0%
Nonirradiated Allograft	788	0.64	0.38 to 1.05	0.08	0%
The type of graft					
Soft Tissue	713	0.49	0.29 to 0.81	0.006	0%
BPTB	456	0.35	0.19 to 0.65	0.001	27%
**Overall IKDC level**					
Total	1433	1.03	1.00 to 1.06	0.03	0%
Irradiation used or not					
Irradiated Allograft	460	1.05	0.99 to 1.12	0.08	0%
Nonirradiated Allograft	973	1.02	0.99 to 1.05	0.19	0%
The type of graft					
Soft Tissue	977	1.02	0.99 to 1.06	0.20	0%
BPTB	456	1.05	1.00 to 1.10	0.05	0%
**Lysholm score**					
Total	1437	0.27	-0.79 to 1.32	0.62	59%
Irradiation used or not					
Irradiated Allograft	460	2.56	1.02 to 4.10	0.001	0%
Nonirradiated Allograft	977	-0.77	-1.37 to -0.17	0.01	45%
The type of graft					
Soft Tissue	875	-0.12	-0.85 to 0.62	0.76	45%
BPTB	562	0.18	-2.01 to 2.36	0.87	74%
**Pivot-shift test**					
Total	1538	1.06	1.02 to 1.11	0.008	50%

Irradiation used or not					
Irradiated Allograft	459	1.21	1.04 to 1.40	0.01	65%
Nonirradiated Allograft	1079	1.01	0.96 to 1.06	0.68	0%
The type of graft					
Soft Tissue	977	1.04	0.96 to 1.13	0.30	52%
BPTB	561	1.09	0.98 to 1.20	0.11	52%
**Lachman test**					
Total	1154	1.14	1.02 to 1.28	0.02	65%
Irradiation used or not					
Irradiated Allograft	459	1.48	1.08 to 2.03	0.02	82%
Nonirradiated Allograft	695	1.03	0.95 to 1.12	0.45	0%
The type of graft					
Soft Tissue	593	1.22	1.00 to 1.48	0.05	65%
BPTB	561	1.09	0.94 to 1.27	0.26	69%
**Tegner score**					
Total	945	0.26	0.06 to 0.45	0.01	0%
Irradiation used or not					
Irradiated Allograft	259	0.57	0.23 to 0.91	0.001	0%
Nonirradiated Allograft	686	0.10	-0.14 to 0.34	0.41	0%
The type of graft					
Soft Tissue	690	0.24	0.02 to 0.46	0.04	0%
BPTB	255	0.31	-0.09 to 0.71	0.13	0%
**Instrumented laxity test**					
Total	919	-0.74	-1.15 to -0.32	0.0005	89%
Irradiation used or not					
Irradiated Allograft	180	-2.45	-3.72 to -1.19	0.0001	79%
Nonirradiated Allograft	739	-0.10	-0.22 to 0.01	0.08	0%
The type of graft					
Soft Tissue	664	-0.83	-1.41 to -0.25	0.005	90%
BPTB	255	-0.81	-1.72 to 0.09	0.08	90%

**Subjective IKDC score**					
Total	1057	1.51	-0.13 to 3.14	0.07	72%
Irradiation used or not					
Irradiated Allograft	180	3.79	1.61 to 5.98	0.0007	0%
Nonirradiated Allograft	877	0.66	-1.24 to 2.56	0.50	77%
The type of graft					
Soft Tissue	696	1.32	-1.33 to 3.96	0.33	70%
BPTB	361	2.15	1.31 to 2.99	< 0.00001	0%
**Daniel 1-leg hop test**					
Total	788	0.99	0.91 to 1.07	0.81	72%
Irradiation used or not					
Irradiated Allograft	396	0.96	0.79 to 1.17	0.70	87%
Nonirradiated Allograft	392	1.01	0.96 to 1.06	0.72	0%
The type of graft					
Soft Tissue	332	1.03	0.97 to 1.09	0.38	0%
BPTB	456	0.95	0.80 to 1.12	0.52	87%

IKDC = International Knee Documentation Committee, BPTB = bone-patellar tendon-bone.

Supplementary Table S4 Sensitivity analyses

Sensitivity analysis	Sample size	Measures of effects size and precision			Heterogeneity
		Point estimate	95% CI	P value	I^2
**Clinical failure**					
All studies (RR)	1169	0.42	0.28 to 0.63	< 0.0001	0%
All studies (OR)	1169	0.38	0.25 to 0.59	< 0.0001	7%
Excluding the largest trial	968	0.49	0.32 to 0.76	0.002	0%
Excluding the most weighted trial	968	0.49	0.32 to 0.76	0.002	0%
Excluding Bottoni 2015	1072	0.44	0.29 to 0.68	0.0002	0%
Excluding Lawhorn 2012	1067	0.41	0.27 to 0.61	< 0.0001	0%
Excluding Sun 2009a1	1119	0.41	0.28 to 0.62	< 0.0001	2%
Excluding Sun 2009a2	1120	0.45	0.30 to 0.67	0.0001	0%
Excluding Sun 2009b	1013	0.38	0.25 to 0.59	< 0.00001	0%
Excluding Sun 2011a	1102	0.45	0.30 to 0.68	0.0002	0%
**Overall IKDC level**					
All studies (RR)	1433	1.03	1.00 to 1.06	0.03	0%
All studies (OR)	1433	1.58	1.04 to 2.40	0.03	0%
Excluding the largest trial	1151	1.04	1.00 to 1.07	0.04	0%
Excluding the most weighted trial	1151	1.04	1.00 to 1.07	0.04	0%
Excluding Bi 2013	1354	1.03	1.00 to 1.06	0.03	0%
Excluding Lawhorn 2012	1331	1.04	1.00 to 1.07	0.02	0%
Excluding Sun 2009a1	1383	1.03	1.00 to 1.06	0.03	0%
Excluding Sun 2009a2	1384	1.03	1.00 to 1.06	0.04	0%
Excluding Sun 2009b	1277	1.03	1.00 to 1.07	0.03	0%
Excluding Sun 2011a	1366	1.03	1.00 to 1.06	0.04	0%
**Lysholm score**					
All studies (MD)	1437	0.27	-0.79 to 1.32	0.62	59%

All studies (SMD)	1437	0.03	-0.14 to 0.20	0.73	59%
Excluding the largest trial	1155	0.51	-0.80 to 1.82	0.45	62%
Excluding the most weighted trial	1155	0.51	-0.80 to 1.82	0.45	62%
Excluding Bi 2013	1358	0.17	-0.92 to 1.25	0.76	61%
Excluding Sun 2009a1	1387	0.33	-0.77 to 1.43	0.55	63%
Excluding Sun 2009a2	1388	0.18	-0.89 to 1.25	0.74	61%
Excluding Sun 2009b	1281	0.44	-0.71 to 1.60	0.45	62%
Excluding Sun 2011a	1370	0.20	-0.88 to 1.29	0.71	61%
**Pivot-shift test**					
All studies (RR)	1538	1.06	1.02 to 1.11	0.008	50%
All studies (OR)	1538	1.45	1.10 to 1.92	0.008	26%
Excluding the largest trial	1256	1.07	1.01 to 1.15	0.03	51%
Excluding the most weighted trial	1256	1.07	1.01 to 1.15	0.03	51%
Excluding Bi 2013	1459	1.06	0.99 to 1.14	0.07	54%
Excluding Lawhorn 2012	1436	1.07	1.02 to 1.12	0.005	52%
Excluding Sun 2009a1	1488	1.06	1.00 to 1.13	0.07	54%
Excluding Sun 2009a2	1489	1.05	1.01 to 1.10	0.03	35%
Excluding Sun 2009b	1382	1.07	1.00 to 1.14	0.06	53%
Excluding Sun 2011a	1471	1.05	1.00 to 1.09	0.04	34%
**Lachman test**					
All studies (RR)	1154	1.14	1.02 to 1.28	0.02	65%
All studies (OR)	1154	1.84	1.18 to 2.85	0.007	53%
Excluding the largest trial	954	1.17	1.02 to 1.34	0.03	69%
Excluding the most weighted trial	954	1.17	1.02 to 1.34	0.03	69%
Excluding Bi 2013	1075	1.15	1.02 to 1.30	0.03	69%
Excluding Sun 2009a1	1104	1.16	1.02 to 1.31	0.02	69%
Excluding Sun 2009a2	1105	1.10	1.03 to 1.18	0.003	48%
Excluding Sun 2009b	998	1.17	1.03 to 1.34	0.02	68%

Excluding Sun 2011a	1087	1.09	1.03 to 1.17	0.007	47%
**Tegner score**					
All studies (MD)	945	0.26	0.06 to 0.45	0.01	0%
All studies (SMD)	945	0.16	0.03 to 0.28	0.02	0%
Excluding the largest trial	759	0.29	0.07 to 0.50	0.008	0%
Excluding the most weighted trial	759	0.29	0.07 to 0.50	0.008	0%
Excluding Bi 2013	866	0.20	-0.01 to 0.41	0.06	0%
Excluding Bottoni 2015	848	0.25	0.05 to 0.45	0.01	0%
Excluding Sun 2009a1	895	0.26	0.06 to 0.46	0.01	0%
Excluding Sun 2009a2	896	0.23	0.03 to 0.43	0.02	0%
Excluding Sun 2009b	789	0.26	0.06 to 0.47	0.01	0%
Excluding Sun 2011a	878	0.22	0.02 to 0.43	0.03	0%
**Instrumented laxity test**					
All studies (MD)	919	-0.74	-1.15 to -0.32	0.0005	89%
All studies (SMD)	919	-0.49	-0.81 to -0.17	0.002	80%
Excluding the largest trial	637	-0.98	-1.54 to -0.42	0.0006	89%
Excluding the most weighted trial	637	-0.98	-1.54 to -0.42	0.0006	89%
Excluding Sun 2009a1	869	-0.86	-1.34 to -0.39	0.0004	90%
Excluding Sun 2009a2	870	-0.52	-0.88 to -0.15	0.006	86%
Excluding Sun 2009b	763	-0.94	-1.47 to -0.41	0.0005	90%
Excluding Sun 2011a	852	-0.46	-0.80 to -0.12	0.008	83%
**Subjective IKDC score**					
All studies (MD)	1057	1.51	-0.13 to 3.14	0.07	72%
All studies (SMD)	1057	0.24	-0.02 to 0.50	0.07	75%
Excluding the largest trial	775	2.21	1.44 to 2.98	< 0.00001	0%
Excluding the most weighted trial	951	1.36	-0.58 to 3.29	0.17	58%
Excluding Bottoni 2015	960	1.47	-0.22 to 3.15	0.09	75%

Excluding Sun 2009a1	1007	1.66	-0.05 to 3.37	0.06	75%
Excluding Sun 2009a2	1008	1.39	-0.31 to 3.08	0.11	75%
Excluding Sun 2009b	901	1.45	-0.37 to 3.28	0.12	75%
Excluding Sun 2011a	990	1.30	-0.41 to 3.01	0.14	74%
**Daniel 1-leg hop test**					
All studies (RR)	788	0.99	0.91 to 1.07	0.81	72%
All studies (OR)	788	0.64	0.40 to 1.00	0.05	48%
Excluding the largest trial	587	1.02	0.98 to 1.07	0.31	0%
Excluding the most weighted trial	632	0.98	0.89 to 1.09	0.76	77%
Excluding Bi 2013	709	0.98	0.89 to 1.08	0.70	77%
Excluding Sun 2009a1	738	0.98	0.89 to 1.08	0.75	76%
Excluding Sun 2009a2	739	0.98	0.90 to 1.08	0.71	77%
Excluding Sun 2009b	632	0.98	0.89 to 1.09	0.76	77%
Excluding Sun 2011a	721	0.98	0.89 to 1.07	0.61	75%

IKDC = International Knee Documentation Committee, CI = confidence interval, RR = relative risk, OR = odds ratio, MD = mean difference, SMD = standardized mean difference.
